# Bed Rest and Hypoxic Exposure Affect Sleep Architecture and Breathing Stability

**DOI:** 10.3389/fphys.2017.00410

**Published:** 2017-06-20

**Authors:** Shawnda A. Morrison, Dani Mirnik, Spela Korsic, Ola Eiken, Igor B. Mekjavic, Leja Dolenc-Groselj

**Affiliations:** ^1^Department of Automation, Biocybernetics and Robotics, Jožef Stefan InstituteLjubljana, Slovenia; ^2^Faculty of Health Sciences, University of PrimorskaIzola, Slovenia; ^3^Division of Neurology, Institute of Clinical Neurophysiology, University Medical CentreLjubljana, Slovenia; ^4^Department of Environmental Physiology, Swedish Aerospace Physiology Centre, Royal Institute of TechnologyStockholm, Sweden; ^5^Department of Biomedical Physiology and Kinesiology, Simon Fraser UniversityBurnaby, BC, Canada

**Keywords:** hypoxia, periodic breathing, high altitude, polysomnography, duty ratio

## Abstract

**Objective:** Despite over 50 years of research on the physiological effects of sustained bed rest, data characterizing its effects on sleep macrostructure and breathing stability in humans are scarce. This study was conducted to determine the effects of continuous exposure to hypoxia and sustained best rest, both individually and combined, on nocturnal sleep and breathing stability.

**Methods:** Eleven participants completed three randomized, counter-balanced, 21-days trials of: (1) normoxic bed rest (NBR, P_I_O_2_ = 133.1 ± 0.3), (2) hypoxic ambulatory confinement (HAMB, P_I_O_2_ = 90.0 ± 0.4) and (3) hypoxic bed rest (HBR, P_I_O_2_ = 90.0 ± 0.4; ~4,000 m equivalent altitude). Full objective polysomnography was performed at baseline, on Night 1 and Night 21 in each condition.

**Results:** In NBR Night 1, more time was spent in light sleep (10 ± 2%) compared to baseline (8 ± 2%; *p* = 0.028); Slow-wave sleep (SWS) was reduced from baseline in the hypoxic-only trial by 18% (HAMB Night 21, *p* = 0.028) and further reduced by 33% (HBR Night 1, *p* = 0.010), and 36% (HBR Night 21, *p* = 0.008) when combined with bed rest. The apnea-hypopnea index doubled from Night 1 to Night 21 in HBR (32–62 events·h^−1^) and HAMB (31–59 events·h^−1^; *p* = 0.002). Those who experienced greatest breathing instability from Night 1 to Night 21 (NBR) were correlated to unchanged or higher (+1%) night SpO_2_ concentrations (*R*^2^ = 0.471, *p* = 0.020).

**Conclusion:** Bed rest negatively affects sleep macrostructure, increases the apnea-hypopnea index, and worsens breathing stability, each independently exacerbated by continuous exposure to hypoxia.

## Introduction

Sleep, defined as a periodic and reversible state of being (Berry et al., 2016), is critical for rest, repair and survival of a species (Hardin, 2009). Voluntary respiratory control becomes absent during sleep, and hypoxic and hypercapnic ventilatory drive is reduced compared to wakefulness (Douglas, 2005). Poor sleep is frequently reported when patients are confined to their bed for extended periods of time, e.g., in intensive care unit (ICU) settings, where sleep disturbances are reported in up to 50% of critically ill patients (Walder et al., 2007). Polysomnography has verified alterations in sleep architecture, decreased total sleep time, and sleep fragmentation in ICU patients (Buckle et al., 1992). These disruptions are often attributed to noise, interruptions to complete diagnostic tests, or other environmental factors (Freedman et al., 1999). Healthy subjects sleep-deprived 24–30-h can experience 17–24% decreases in ventilatory responsiveness to hypercapnia, and increased respiratory muscle fatigue (Chen and Tang, 1989). Evidence alludes to prolonged time spent in bed altering patients' ventilatory drive; sleep deprivation *per se* may play a role in ventilatory chemoreceptor mechanisms, although these findings are not universally supported (Spengler and Shea, 2000). A clinical example includes chronic obstructive pulmonary disease, which is a frequent cause of ICU admission (Faisy et al., 2016), however little is known about the respiratory pattern in patients experiencing prolonged mechanical ventilation in combination with prolonged bed rest. These patients usually remain in the ICU up to 30 days, although chronic alterations can be observed in as little as 3 weeks' duration (Chlan et al., 2015).

Determining whether bed rest and subsequent sleep disturbances lead to poor ventilatory control in ICU patients can be especially difficult since critical care patients often exhibit complicated co-morbidities. Confining otherwise healthy humans to bed rest is a relatively common experimental model in space life-sciences, used as a ground-based analog to induce similar physiological strain as experienced in microgravity (reviewed in Pavy-Le Traon et al., 2007). Yet despite over 50 years of bed-rest studies in humans, there is very little objectively-measured sleep data (Pavy-Le Traon et al., 2007). Of the few experimental studies investigating any sleep measure, many have not included baseline night recordings (Komada et al., 2006) or are confounded by experimental designs which manipulate circadian rhythm or light exposure (Monk et al., 1997), directly affecting sleep data. Thus, the effect of sustained bed rest on objective sleep parameters in healthy humans is not well known.

Sleep studies conducted in hypoxic environments have reported alterations in sleep architecture, including reductions in slow-wave sleep (SWS), more frequent arousals, and marked periodic breathing (reviewed in Ainslie et al., 2013). Ascent to high altitude in newcomers leads to unstable breathing during both wakefulness and sleep in up to 90% of those venturing above 5,000 m (Burgess et al., 2004), and this breathing instability can persist up to 12 months when living in hypoxic confinement (Tellez et al., 2014). The recurrent hypoxemia and hypercapnia as a consequence of the respiratory pauses in periodic breathing can have extensive adverse health consequences, including decreased neurocognitive function (sleepiness, mood changes, depression), and cardiovascular complications such as pulmonary hypertension, arrhythmias, ischemic heart disease, myocardial infarction, diabetes, and stroke (Peppard et al., 2000; Wolk et al., 2003; Arzt et al., 2005). These abnormal breathing patterns can lead to serious complications for the heart and brain, especially in patient populations who may suffer from temporary or chronic respiratory insufficiency and are thus both hypoxic and physically inactive.

It is important to note that disease-states or high-altitude exposures are not the only scenarios where humans may find themselves exposed to hypoxic conditions for long durations. Indeed, it is envisioned that future planetary habitats will maintain lower partial pressure of oxygen. Data on the interactions between hypoxic exposure and bed rest are scarce, although recent work has investigated the physiological effects of both stressors on body composition (Debevec et al., 2014b), psychological strain of confinement (Stavrou et al., 2015) and sleep architecture (Rojc et al., 2014) in short-term (10-days) exposures. Considering the clinical data on certain patient populations finds significant differences in breathing after 3 weeks, and also international space-science guidelines for moderate-duration bed rest studies, a 21-days exposure was chosen to mimic the conditions of prolonged immobility and hypoxia.

The purpose of this study was to determine the separate and combined effects of a 21-days duration bed rest and hypoxic exposure on nocturnal sleep macrostructure and respiratory outcomes. It was hypothesized that sleep would be more fragmented at the start of bed rest under normal (normoxic) conditions, but that hypoxia would induce significant, negative alterations in respiratory control compared to baseline recordings, and sleep quality and breathing stability issues would be exacerbated when both stimuli were combined.

## Materials and methods

This sleep study was part of a larger investigation exploring the separate and combined effects of inactivity/unloading and hypoxia on several physiology systems. Specific data related to appetite (Debevec et al., 2016), insulin sensitivity (Simpson et al., 2016), bone health (Rittweger et al., 2016), hematology (Keramidas et al., 2016b), peak oxygen uptake (Keramidas et al., 2016a), and skeletal muscle miRNA expression (Rullman et al., 2016), are available elsewhere.

### Study participants

All procedures performed were in accordance with the ethical standards of the institutional and national research committee and with the 1964 Helsinki declaration and its later amendments. This study was approved by the Ministry of Health of the Republic of Slovenia and the National Committee for Medical Ethics, (#205/02/11). Informed consent was obtained from all individual participants prior to inclusion in the study.

All testing was performed at the Olympic Sports Centre Planica (Rateče, Slovenia) situated 940 m above sea level. Sixty-five males were initially screened for inclusion into the study. Following preliminary testing, including a supervised, overnight familiarization weekend at the hypoxic facility, 14 participants were selected and invited to participate in the study. Eleven participants completed all testing trials and their details are reported herein. Exclusion criteria followed international standards for bed rest investigations (Standardization of bed rest study conditions, Version 1.5, August 2009). Participants were excluded if they reported: any underlying medical conditions, were smokers, were uncomfortable remaining within the hypoxic facility, or were unable to commit to completing three full trials. Thus, all participants were non-smokers, sea-level dwellers, with no history of high-altitude exposures within the past 4 months, no known cardiorespiratory, musculoskeletal, asthma, allergy, or circulatory disease.

In the final trial, two participants did not return because of employment; one participant was withdrawn due to gastrointestinal issues (unrelated to the bed rest procedure). Eleven of 14 participants completed all three trials and are included in all subsequent analyses (20–41 years). This sleep and respiration study was part of a larger investigation exploring the separate and combined effects of simulated low-gravity (bed rest) and hypoxic exposure on various aspects of human physiology.

### Study design and hypoxic environment

**Figure 1:**
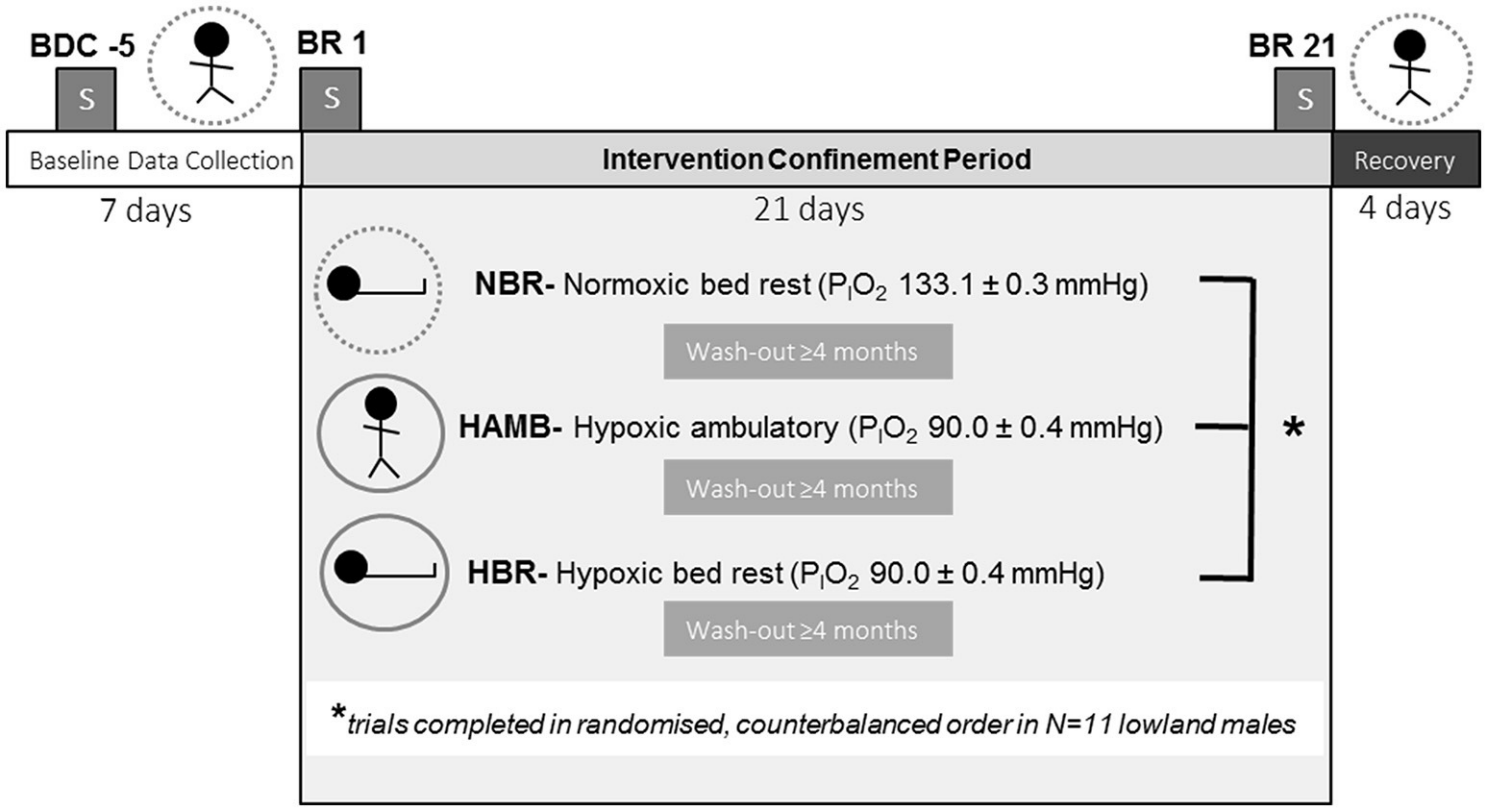
Experimental protocol. BDC, baseline data collection; BR, bed rest; NBR, normoxic bed rest; HAMB, hypoxic ambulatory; HBR, hypoxic bed rest confinement conditions. Symbols for each experimental condition include: dashed circle (normobaric normoxia), or solid circle (normobaric hypoxia), and the subject either upright (ambulatory) or supine (bed rest). Blocked “S” symbol indicates on which night(s) sleep PSG measurements were conducted.

Participants entered the experimental trial in pairs. Each trial had a total duration of 32-days. Initial baseline testing was 7-days duration, during which participants engaged in a host of experimental procedures, including assessments of: body composition, aerobic fitness, bone mineral density, orthostatic tolerance, muscle physiology (isokinetic dynamometry, muscle biopsy), behavioral thermoregulation, sleep, and others. Baseline testing was immediately followed by a 21-days intervention (i.e., a medium-duration bed-rest protocol according to European Space Agency and National Aeronautics and Space Agency standards) in a randomized, counterbalanced, repeated-measures fashion (NBR, HAMB, or HBR), with a final 4-days recovery phase, during which all post-testing measurements were completed (Figure 1). Participants remained confined to the testing facility for the entire experimental session (thus the 32-days total confinement at the facility). When the participants were not in the 21-days intervention period, they were permitted short, supervised outings to a picnic area around the immediate building site. Due to the repeated-measures aspect of testing, each subject acted as their own control, with baseline testing constituting the normal “control” dataset when they were both normoxic and ambulatory. Subsequent experimental conditions were balanced to determine the effect of minimal activity (bed rest), or hypoxic exposure, or the effect of both stimuli combined. Specifically, the experimental conditions were: (1) normoxic bed rest (NBR, inspired partial pressure of oxygen, P_I_O_2_ = 133.1 ± 0.3), (2) hypoxic ambulatory (HAMB, P_I_O_2_ = 90.0 ± 0.4), and (3) hypoxic bed rest (HBR, P_I_O_2_ = 90.0 ± 0.4, ~4,000 m equivalent altitude). For safety reasons, and because the physical impact of remaining in such a hypoxic environment is very obvious, neither the subjects nor the researchers were blinded regarding their breathing gas. There was a 4-month wash-out period between trials to ensure adequate physiological and psychological recovery. The normobaric hypoxia was maintained by a vacuum pressure swing absorption system (b-Cat, Tiel, The Netherlands), described in detail elsewhere (Debevec et al., 2014a). Oxygen levels in the common area and individual rooms were continuously monitored with O_2_ sensors (PGM-1100; Rae Systems, San Jose, CA).

### Daily experimental test protocols

Participants were awakened at 7:00 and lights turned off at 23:00 each day throughout the experiment. Napping was prohibited. Resting heart rate and peripheral SpO_2_ were measured each morning (short-range telemetry, iBody, Wahoo Fitness, Atlanta, USA, 3100 WristOx, Nonin Medicals, Minnesota, USA, respectively). Participants then completed the various tests they had scheduled for that day, or engaged in sedentary leisure activities (e.g., reading, watching movies, studying, visiting the other subjects) whilst waiting for the next examination. Participants filled in the self-assessment portion of the Lake Louise questionnaire score (LLS) at 17:00 each evening. Diagnoses of acute mountain sickness (AMS) were defined as: (1) LLS ≥3 and (2) presence of headache.

Participants were confined to a horizontal position during both NBR and HBR following established protocols (Pavy-Le Traon et al., 2007). All activities of daily living (e.g., eating, reading, and showering) were carried out in the horizontal position. Participants used one pillow for head support. They were allowed to drink water *ad libitum*, and were actively encouraged to drink at least 2 L per day. Five meals were served daily (breakfast, morning snack, lunch, afternoon snack and dinner), and always at the same time of day throughout each trial. Participants were encouraged to consume all food provided; they could opt to consume less, but they did not receive any additional food than the original prescribed amounts. A 14-day menu was used during the first trial and rotated throughout the trial duration. This same menu was then applied to the two subsequent trials. Thus, participants consumed identical meals, and on the same testing day(s), of each trial. The menu was designed using an in-house web-based application (OPKP, Jozef Stefan Institute, Ljubljana, Slovenia), consisting of targeted baseline macronutrient compositions of ~55% carbohydrate, ~30% fat and ~15% protein. Alcohol and caffeine were not included in the standardized diet. A detailed summary of daily nutritional protocols (including sample menus) are available elsewhere (Debevec et al., 2014a).

During HAMB, participants were encouraged to move about the common hypoxic living area (~200 m^2^) and maintain an upright (standing) position. To mimic unconfined activity levels participants performed two structured, 30-min low-intensity exercise sessions each day, once in the morning and once in the afternoon. Exercise mode varied with each session (stepping, cycling or activity-based video games) to avoid monotony. During all sessions, HR and pulsed oximetry were monitored to ensure the individual's intensity was maintained within the targeted value (~123 ± 4 beats·min^−1^), roughly corresponding to 50% of the participants' hypoxic-specific peak power output determined previously from a hypoxic graded exercise test (Debevec et al., 2014a). The graded test was performed before confinement on a cycle ergometer under hypoxic condition (F_I_O_2_ = 0.144) using 25 W·min^−1^ workload increments until task failure, defined as an inability to maintain cycling cadence >60 rotations per minute. Activity-based video games and table football were also provided to promote upright daily activity.

### Night polysomnography

Full ambulatory polysomnography (PSG, Nicolet One, Viasys, Healthcare, Neurocare, Madison, WI, USA) was performed using standard set-ups (Burgess et al., 2004; Ainslie et al., 2007; Rojc et al., 2014). This included PSG recordings of: electroencephalography (EEG), electro-oculography (EOG), chin and tibial surface electromyography (EMG), electrocardiography (ECG), nasal pressure (nasal pressure cannula), respiratory movements (chest and abdominal belts), and capillary oxyhaemoglobin saturation.

Continuous video surveillance during the night was implemented for subjects' safety, and to monitor subjects' movements. Testing occurred after subjects had spent at least two nights in the testing facility; during each trial, measurements were conducted on three occasions (1) baseline data collection day 3 (i.e., the “control” data), (2) Night 1, and (3) Night 21 of the intervention.

All recordings were visually scored and analyzed by a certified sleep physician from an accredited sleep laboratory, based on the American Academy of Sleep Medicine (AASM) manual for the scoring of sleep and associated events (Berry et al., 2016) (detailed below). Participants were excluded from the sleep study if it was discovered they had periodic limb movements (PLM index > 5/h), obstructive sleep apnea (apnea-hypopnea index, AHI >5), or any other sleep abnormalities.

### Data analysis and statistical measures

Duty ratio (DR) was calculated as a surrogate for loop gain (Edwards et al., 2008; Sands et al., 2011), using methods described elsewhere (Andrews et al., 2012). Briefly, apneas were scored as a drop in the peak signal excursion by ≥90% of pre-event baseline using the oro-nasal sensor for ≥10 s with associated absent inspiratory effort throughout the entire period of absent airflow, and hypopneas scored as a peak signal excursion drop by ≥30% of pre-event baseline using nasal pressure for ≥10 s with a ≥3% oxygen desaturation from pre-event baseline, unless the apneas or hypopneas occurred as a segment of continuous periodic breathing, in which case the entire duration of the respiratory event was taken. DR was calculated as mean time (in seconds) of hyperpnea/(hyperpnea + apnea) (Edwards et al., 2008; Andrews et al., 2012).

On all dependent variables, a 2-way repeated measures ANOVA was conducted with two between subjects' factors [time, (Baseline, Night 1, Night 21)] and condition (NBR, HBR, HAMB) and paired *t*-tests employed *post-hoc* (Bonferroni correction). Bivariate correlations were run between variables of interest; statistical analyses were limited to reduce the likelihood of Type I error, at an alpha level of 0.05. Data were analyzed using SPSS (v.20.0, IBM Statistics, Chicago, IL, USA) and expressed as means ± standard deviations in all text and figures.

## Results

### General environmental adaptations

One subject experienced severe hypoxemia (SpO_2_ <75%) combined with dizziness and headache during the first hours of his HBR intervention. He was relocated to a separate room where the simulated altitude was 3,000 m, during which the symptoms subsided. The following day, the simulated altitude was increased to 3,500 m, and finally on the third day to 4,000 m, with no further difficulties. This ascent profile was repeated for his HAMB condition. All sleep-breathing parameters for this subject were within one standard deviation of group means, thus his data are included in all further analyses.

For all subjects, daily morning SpO_2_ was lower in HAMB (88 ± 1%) and HBR (88 ± 2%) compared to NBR (97 ± 0%) during the 21-days intervention (*p* < 0.01). There was a gradual recovery of SpO_2_ following the first day in hypoxia, such that SpO_2_ was higher in HBR from day 3–21 (89 ± 1%), and in HAMB from 16 to 21 (89 ± 0%) compared to the first day in normobaric hypoxia (85 ± 3%; *p* < 0.05). Supine morning HR was higher in HBR (74 ± 9 b·min^−1^) than in NBR (60 ± 10 b·min^−1^) from days 14 to 21 (*p* < 0.05). Average HR and SpO_2_ responses to the low-intensity physical activity sessions performed during HAMB were 124 ± 9 b·min^−1^ and 87 ± 3%, respectively.

### Sleep architecture and quality

**Figure 2:**
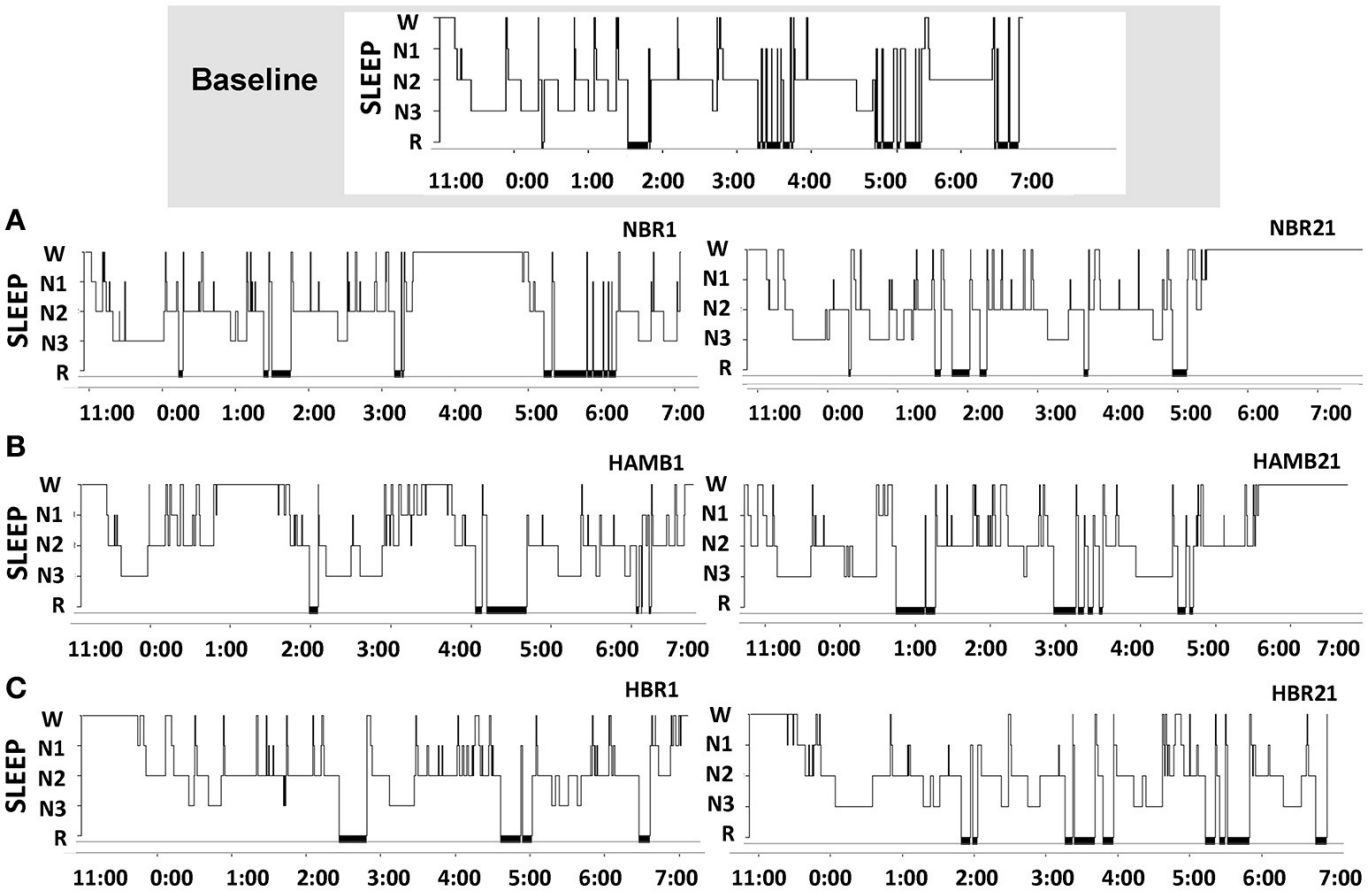
Sleep architecture changes across the protocol in one representative subject for the baseline control night, then **(A)** normoxic bed rest night one (NBR1) and normoxic bed rest night 21 (NBR21), **(B)** hypoxic ambulatory night one (HAMB1) and hypoxic ambulatory night 21 (HAMB21), **(C)** hypoxic bed rest night one (HBR1) and hypoxic bed rest night 21 (HBR21). SLEEP, sleep stages (N1, N2, N3, R); x-axis denote time of night in 24-h clock (lights out 23:00, lights on 07:00).

Sleep architecture responses to all experimental conditions are displayed for one representative subject in Figure 2. In terms of mean group data, participants spent more time in N1 during NBR Night 1 compared to the baseline control night (Baseline: 8 ± 2, NBR Night 1: 10 ± 2%; *p* = 0.028). There were significant differences between NBR and HBR conditions (95% CI: −4.4 to −0.2%; *p* = 0.028), irrespective of time. After 21-days in hypoxia, subjects continued to experience a greater proportion of time spent in N1 (HAMB Night 21, 11 ± 4%, *p* = 0.018, HBR Night 21, 11 ± 3% *p* = 0.031, Table 1). After 21-days of bedrest (both NBR and HBR), the proportion of R was increased 17.8 ± 4.3% compared to the baseline control night (NBR Night 21 95% CI: 9.7–11.8% *p* = 0.017; HBR Night 21 95% CI: 8.1–13.3%, *p* = 0.012). Thus, sleep architecture was altered by bed rest alone, and the continuous bed rest plus hypoxic exposure combination.

**Table 1:** Polysomnography sleep data for the baseline control night, Night 1 and Night 21 experimental trials, including the normoxic bed rest (NBR), hypoxic ambulatory (HAMB) and hypoxic bed rest (HBR) conditions.

**Sleep Variables**	**Baseline**	**Night 1**			**Night 21**		
		**NBR**	**HAMB**	**HBR**	**NBR**	**HAMB**	**HBR**
**SLEEP ARCHITECTURE**							
Total Sleep Time, h	7.3 ± 0.6	7.2 ± 0.7	7.3 ± 0.5	6.8 ± 0.8^*^	6.7 ± 0.8^*^	7.2 ± 0.4	6.8 ± 0.5^*^
	(6.3–7.8)	(5.7–8.1)	(6.0–7.8)	(4.8–7.8)	(5.5–7.6)	(6.6–7.7)	(5.7–7.3)
N1, %	7.7 ± 2.1	9.7 ± 2.4^*^	9.3 ± 4.0	11.8 ± 3.3^*^	8.1 ± 2.8^†^	10.8 ± 3.6^*^	10.6 ± 3.3^*^
	(5.0–12.0)	(7.0–14.0)	(5.0–15.0)	(9.0–21.0)	(4.0–13.0)	(6.0–19.0)	(6.0–17.0)
N2, %	52.3 ± 4.1	51.8 ± 4.6	51.5 ± 8.1	57.0 ± 3.4	49.3 ± 4.0^*^	50.0 ± 5.1	50.2 ± 5.2
	(46.0–59.0)	(43.0–58.0)	(38.0–68.0)	(52.0–64.0)	(43.0–57.0)	(43.0–60.0)	(41.0–59.0)
N3, %	22.2 ± 4.3	19.2 ± 5.4^§^	20.1 ± 6.8	17.1 ± 4.1^*^	19.1 ± 6.1^§^	18.2 ± 5.7^*^	16.5 ± 5.7^*^
	(17.0–31.0)	(10.0–30.0)	(8.0–30.0)	(12.0–25.0)	(9.0–28.0)	(9.0–26.0)	(8.0–27.0)
R, %	17.8 ± 4.3	19.4 ± 5.2^§^	17.5 ± 3.7	16.5 ± 5.0^*^	23.3 ± 6.1^†^	20.9 ± 5.2^*^	22.5 ± 4.0^*^^†^
	(11.0–26.0)	(10.0–28.0)	(11.0–24.0)	(11.0–29.0)	(13.0–32.0)	(10.0–29.0)	(15.0–28.0)
**SLEEP QUALITY**							
WASO, min	45.5 ± 42.6	51.5 ± 32.6	73.2 ± 43.4	41.0 ± 15.4	40.6 ± 31.6	38.2 ± 19.2^†^	42.6 ± 23.7
	(17.0–150.0)	(15.5–108.0)	(7.0–140.0)	(21.0–68.5)	(11.0–107.5)	(6.5–75.5)	(17.0–81.0)
Sleep Latency, min	33.1 ± 31.4	24.8 ± 18.5	16.7 ± 7.4	33.0 ± 15.5	30.7 ± 31.9	30.6 ± 26.8	33.4 ± 19.8
	(5.0–91.0)	(5.0–64.5)	(5.0–31.0)	(8.5–54.5)	(7.5–115.5)	(2.0–99.0)	(10.5–65.5)
REM Latency, min	85.7 ± 30.2	84.2 ± 40.1	111 ± 58	99.3 ± 42.7	76.1 ± 41.3	86.2 ± 38.3	90.0 ± 40.7
	(49.5–156.5)	(33.0–163.5)	(21.5–205.5)	(28.5–162.0)	(36.0–192.5)	(46.5–154.5)	(29.0–145.5)
Sleep Efficiency, %	83 ± 12	82 ± 6	78 ± 9	82 ± 9	79 ± 9	84 ± 7	83 ± 7
	(58–94)	(71–93)	(68–94)	(59–90)	(65–93)	(73–94)	(70–89)
Awakenings, #	26 ± 9	25 ± 4	28 ± 10	27 ± 7	22 ± 6	27 ± 8	26 ± 7
	(12–40)	(19–31)	(9–49)	(16–36)	(12–32)	(8–39)	(15–41)
Stage Shifts, #	109 ± 32	109 ± 9	109 ± 29	120 ± 21	92 ± 22	115 ± 26	112 ± 19
	(42–155)	(97–128)	(68–167)	(86–155)	(66–128)	(67–153)	(76–142)

* *Significantly different from Baseline*,

† *significantly different from Night 1 within-condition*.

§ *p-value between 0.05 and 0.08 compared to Baseline. Values are means ± standard deviations (p < 0.05)*.

*Low and high range values are denoted in brackets directly beneath mean scores*.

Wake after sleep onset (WASO) scores were reduced (i.e., improved) from −32 to −5 min after 21-days in any condition (*p* = 0.011). *Post-hoc* testing reveled that these improvements were specifically attributed to ameliorated values in the HAMB condition, which decreased from 73 ± 43 min (HAMB Night 1) to 38 ± 19 min (HAMB Night 21; 95% CI: 8–62 min; *p* = 0.015). Other markers of sleep quality (e.g., number of awakenings, sleep latency, sleep efficiency) remained unchanged between the baseline control night and the other experimental conditions (NBR, HBR, and HAMB). There were no interactions between-conditions present for sleep quality variables.

### Breathing stability, AHI and relationship to night SpO_2_

**Figure 3:**
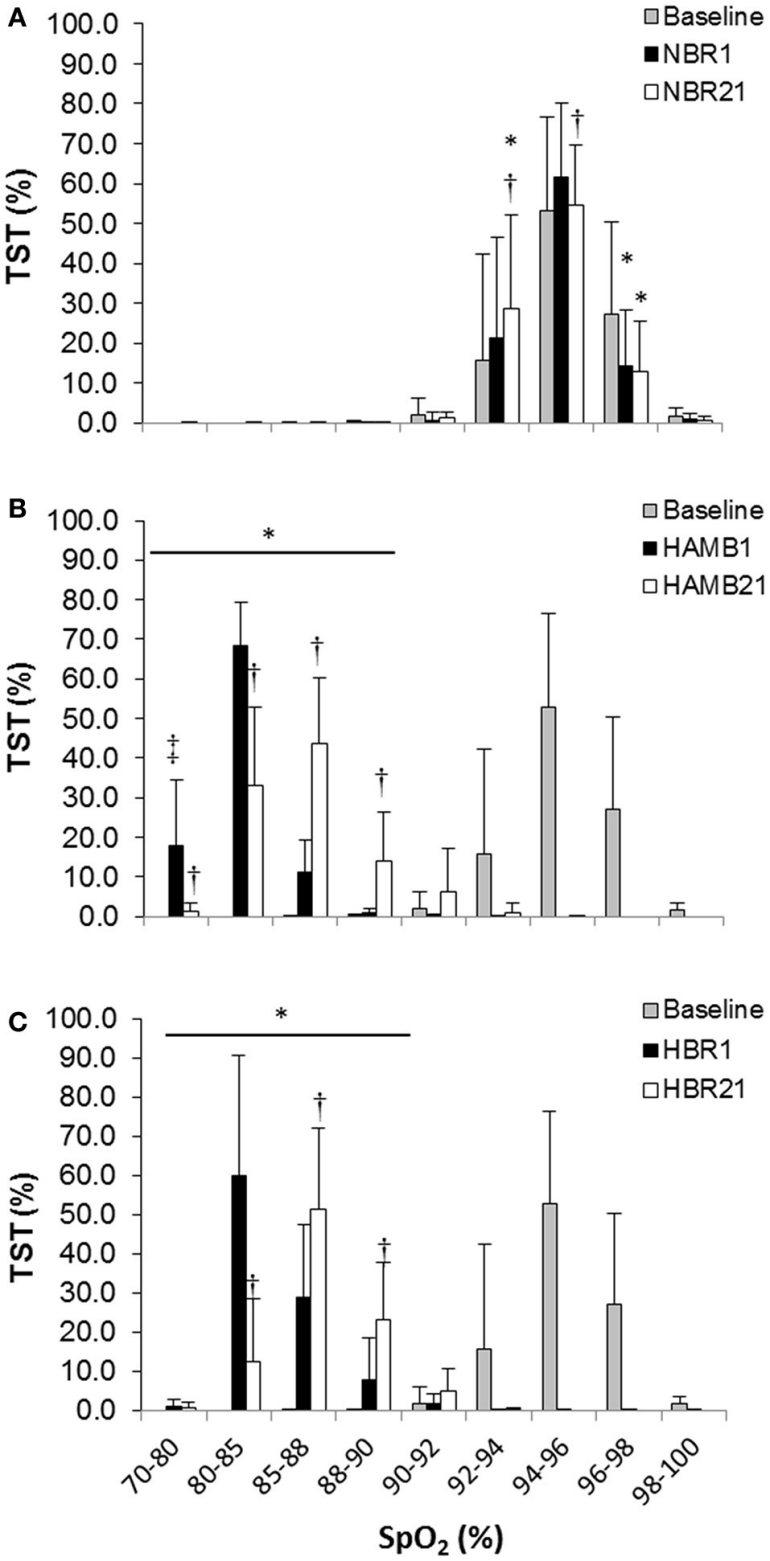
Proportion of total sleep time (TST) spent at a given night peripheral oxyhemoglobin concentration (SpO_2_) for **(A)** normoxic bed rest (NBR), **(B)** hypoxic ambulatory (HAMB) **(C)** hypoxic bed rest (HBR) conditions. Error bars depict standard deviations. (^*^) Significant difference from baseline (all concentrations in both hypoxic trials), (^†^) significant difference from Night 1, within-condition (^‡^) significant difference from all other conditions within-night at that saturation (*p* < 0.05).

**Figure 4:**
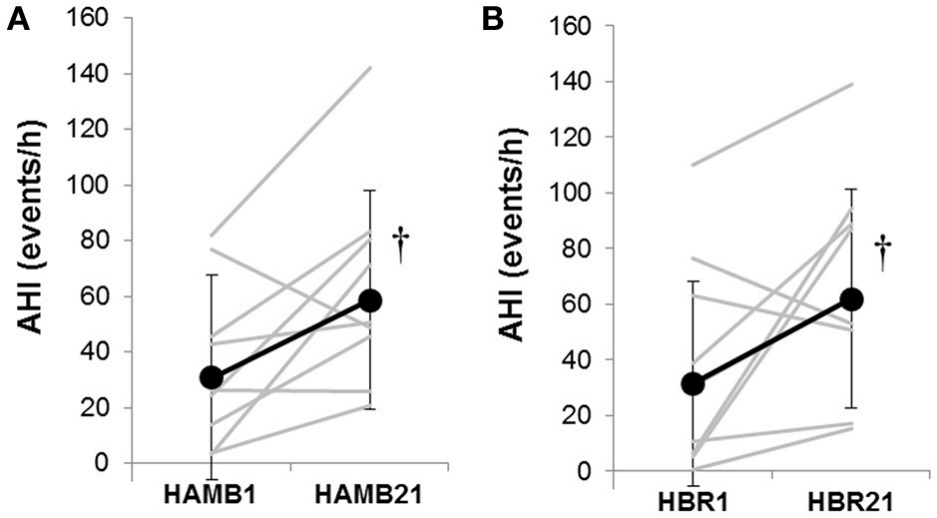
The Apnea-Hypopnea Index (AHI) responses from Night 1 to Night 21 in **(A)** hypoxic ambulatory (HAMB) and **(B)** hypoxic bed rest (HBR) conditions. Individual data are denoted in gray lines; group means are denoted in black lines with closed circles and error bars depicting standard deviations. (^†^) significant increase from Night 1 in both hypoxic conditions (*p* < 0.05).

In both HAMB and HBR, AHI doubled in severity after 21-days of exposure (Night 1: 31 ± 32, Night 21: 60 ± 37 events·h^−1^; *p* = 0.002; Table 2). DRs were 28% lower than baseline (i.e., more unstable) in both hypoxic exposure groups (*p* < 0.001), but were not different between-groups. Breathing stability significantly worsened from NBR Night 1 to NBR Night 21 (CI: −0.283 to −0.093; *p* = 0.001). Mean night SpO_2_ values were affected by hypoxic exposure, rebounding significantly on Night 21, but remaining a full ~5% less than normoxic values (Figure 3). Notably, there were small, but significant decreases in NBR Night 21 compared to NBR Night 1 (95% CI: −1.76 to −0.04%; *p* = 0.041). Changes in AHI from Night 1 to Night 21 (Figure 4) were not correlated to DR or SpO_2_ for any condition (Table 3), whereas changes in DR from NBR Night 1 to Night 21 were significantly correlated to the delta night SpO_2_ change, i.e., the lower the DR, the higher SpO_2_ night concentration (*R*^2^ = 0.4708; *p* = 0.020).

**Table 2:** Sleep-breathing parameters from the Baseline control night, Night 1 and Night 21 of the experimental trials, including the normoxic bed rest (NBR), hypoxic ambulatory (HAMB) and hypoxic bed rest (HBR) conditions.

	**Baseline**	**Night 1**			**Night 21**		
		**NBR**	**HAMB**	**HBR**	**NBR**	**HAMB**	**HBR**
Incidence rate, %	64%	18%	100%	91%	82%	100%	100%
AHI, events.h^−1^	2.1 ± 2.4	0.4 ± 0.8	30.9 ± 28.0^*^	31.5 ± 36.9^*^	4.6 ± 9.0	58.7 ± 36.6^*^^†^	61.8 ± 39.3^*^^†^
Hyperpnea length, s	20.8 ± 5.6	28.6 ± 4.7	14.0 ± 3.8^§^	15.6 ± 3.9^§^	24.7 ± 7.6	14.4 ± 3.1^*^^‡^	15.9 ± 3.6^‡^^§^
Apnea length, s	18.9 ± 4.9	18.1 ± 4.0	11.6 ± 1.8^*^	12.4 ± 1.3^*^	17.0 ± 3.4	11.9 ± 1.4^*^^‡^	12.2 ± 1.9^*^^‡^
Total cycle length (H+A)	39.6 ± 6.7	46.7 ± 8.7	25.6 ± 5.2^*^	28.0 ± 4.6^*^	41.6 ± 10.6^†^	26.3 ± 4.0^*^^‡^	28.0 ± 5.2^*^^‡^
Duty ratio (H/H+A)	0.972 ± 0.040	0.949 ± 0.113	0.687 ± 0.022^*^	0.719 ± 0.096^*^	0.761 ± 0.120^*^^†^	0.687 ± 0.022^*^	0.696 ± 0.018^*^

Incidence Rate refers to whether a person demonstrated any classifiable apneas and/or hypopneas at any point during sleep in their night PSG recordings. This is not indicative of clinically-relevant AHI scores, only whether there were any officially classifiable respiratory events throughout the night

* *Significantly different from baseline*,

† *different from Night 1 within-condition, different from NBR21 within that time-point*.

§ *p-value between 0.05 and 0.07 compared to baseline*.

*Group-wise comparisons between NBR1 and the other time-points were not analyzed because of the lack of incidence rate within that condition (N = 2/11 only). Values are means ± standard deviations (p < 0.05)*.

**Table 3:** Relationships of selected variable change scores from the baseline control night to Night 1, and from Night 1 to Night 21 of the experimental trials, including the normoxic bed rest (NBR), hypoxic ambulatory (HAMB) and hypoxic bed rest (HBR) conditions.

		**Δ from Baseline to Night 1**			**Δ from Night 1 to Night 21**		
		**NBR**	**HAMB**	**HBR**	**NBR**	**HAMB**	**HBR**
Δ DR vs. Δ AHI	*R*^2^	0.198	0.119	0.201	0.017	0.011	0.012
	*p*-value	0.170	0.298	0.167	0.704	0.756	0.751
Δ AHI vs. Δ SpO_2_	*R*^2^	0.404^*^	0.123	0.309	0.168	0.111	0.209
	*p*-value	0.035^*^	0.290	0.076	0.210	0.317	0.158
Δ DR vs. Δ SpO_2_	*R*^2^	0.058	0.017	0.309	0.471^*^	0.009	0.233
	*p*-value	0.478	0.706	0.076	0.020^*^	0.776	0.133

*DR, duty ratio; AHI, apnea-hypopnea index; SpO_2_, peripheral oxyhemoglobin saturation; NBR, normoxic bed rest; HBR, hypoxic bed rest; HAMB, hypoxic ambulatory condition*.

* *Significant correlation (p < 0.05)*.

## Discussion

The present study demonstrates that breathing stability (characterized by the DR) is worsened during 21-days of normoxic bed rest (NBR Night 21), by acute exposure to hypoxia (HAMB Night 1), and by bed rest combined with hypoxia (HBR Night 1). Breathing responses during nocturnal sleep remain poor after 21-days of exposure, whether people remain recumbent or ambulatory. Sleep architecture is affected in all trials, with the most noticeable shifts occurring in the combined HBR condition. There appears to be a direct relationship between breathing stability and mean night SpO_2_ when otherwise healthy adults are confined to bed, echoing published data that have reported ventilatory drive changes in patients (Schiffman et al., 1983; Chen and Tang, 1989), and lower hypoxic chemoresponsiveness in susceptible individuals at high altitude (Nespoulet et al., 2012).

The initial increase in ventilation during exposure to hypoxia (i.e., hypoxic ventilatory response; HVR) is highly variable (Hirshman et al., 1975). Some studies have stratified test populations into “susceptible” and “resistant” phenotypes when characterizing one's predisposition to suffering AMS and high-altitude pulmonary edema (HAPE) (Hohenhaus et al., 1995; Nespoulet et al., 2012). In one investigation, AMS susceptible patients' night SpO_2_ concentrations were ~5% lower than their non-susceptible counterparts, yet these patients experienced significantly fewer AHI events per hour (18 vs. 33 events per hour; *p* = 0.038) (Nespoulet et al., 2012). The present study observed a highly individualized response in breathing stability, especially after 21-days in bed rest, such that those who experienced the greatest change in breathing stability from Night 1 to Night 21 also maintained or increased night SpO_2_ saturations. Greater oscillations in breathing stability may serve to circulate more overall blood flow to cerebral tissues, affecting ventilatory drive, and further optimizing night mean SpO_2_ values (Ainslie et al., 2007, 2013). These findings are in-line with research conducted on normal, healthy subjects who develop periodic breathing at altitude, and in whom one usually finds that mean SpO_2_ is higher than in individuals who do not develop periodic breathing (Ainslie et al., 2013). Of note, DRs are calculated based on the number of apneas and hyperpnea experienced. Thus, although we observed a significant decline in breathing stability in the NBR trial, these data are based on fewer absolute events per hour compared to in the hypoxic trials (either with bed rest or ambulatory). Therefore, the significant decrease in DR and increased total cycle length observed in NBR may be more heavily weighted on less disturbances overall. The relationship between breathing stability and bed rest should continue to be explored in future research.

Respiration can vary markedly throughout each sleep stage, independent of environmental conditions. Sensitivity to high PCO_2_ and low PO_2_ is lowest in REM vs. NREM sleep (Douglas et al., 1982). Transitioning from wakefulness to N1 is characterized by significant decreases in minute ventilation, and generally attributed to variations in both respiratory rate and tidal volume (Kreiger, 1985). Thus, as NREM sleep progresses, hypoventilation, and a 3–7 mm Hg increase in arterial PCO_2_, occurs as a result of: moderated central respiratory drive, upper respiratory muscle relaxation, and increased airway resistance(Kreiger, 1985). Minute ventilation decreases across N2 and N3 sleep, and is characterized by particularly high individual variability observed between subjects' HVR when transitioning between these sleep stages. Although the alterations in sleep architecture observed in the present study may be, predominantly, an after-effect of the frequent periodic breathing experienced from the continuous hypoxic stimulus, it is useful to note that spending more time in a given sleep stage may affect the HVR in its own right.

It is also important to note that sleep-breathing patterns observed during strict bed rest within a clinical population may be affected by additional whole-body challenges not considered in the present work (e.g., increased inflammatory responses, bone frailty, respiratory complications, or certain medications), in which healthy adults were studied. By contrast, this randomized, repeated-measures investigation sought to objectively measure the influence of sustained bed rest *per se*, and in combination with hypoxia, on sleep-breathing parameters, and accordingly, required the participants to adhere to very strict rules, including prohibiting daytime naps. Certainly, these standards would provide a different sleep experience than those of patients who may be bedridden, but are permitted to nap during the day, for example. Other research involving the combined effects of bed rest and hypoxic exposure have been conducted using shorter, 10-days protocols. In one study, Rojc et al. (2014) found that participants (*N* = 10 males) spent ~40% less time in N1 sleep after 10 days in HBR compared to baseline recordings. There were near-universal incidences of periodic breathing after 10 days in the hypoxic trials (HBR Night 10: 100% HAMB Night 10: 80%), although no data were reported for AHI or breathing stability (DR, or other measure), nor were any data reported on whether the observed periodic breathing was related to any other dependent measure. However, it must also be acknowledged that for practical purposes, the present study did not include a 21-days normoxic ambulatory condition (“NAMB”). We considered that the baseline sleep measurement would be a valid assessment of the subjects' normoxic ambulatory responses under resting conditions, but without the confinement aspect. Thus, it cannot be ruled out that the values we might have obtained with an “NAMB” intervention may have differed significantly from baseline values. Consequently, it is conceivable that, at least to some extent, the effects we have attributed to bed rest *per se* or hypoxia *per se* may be confounded by other unidentified factors, like remaining in confinement.

In the current study, AHI doubled in severity in terms of events per hour, during the course of both hypoxic interventions (Figure 4). Data on progressive increases in AHI with partial or full acclimatization to high altitude have been reported in works that encompass hypoxic exposures from 14 days (at 5,050 m) (Andrews et al., 2012) up to 1 year exposure duration on the high Antarctic plateau (~3,000 m altitude at equator) (Tellez et al., 2014). It has been suggested that instability in respiratory control may be dependent on altitude, (Andrews et al., 2012) not just exposure duration. That there were increases observed in DR and AHI during the course of the NBR intervention remains an avenue to be further explored. In terms of the stated hypotheses, this study did find that sleep architecture was negatively altered at the start of bed rest for both NBR and HBR compared to baseline. Hypoxia induced significant, negative alterations in respiratory control, and clinical indexes like the AHI in HAMB and HBR; however there were no appreciable differences in sleep quality variables found in any condition. Breathing stability issues were not systematically exacerbated when both stimuli were combined, i.e., the HBR condition was no worse than HAMB in terms of AHI, DR or length of the breathing cycle. These sleep data have implications for clinical populations who are bedridden, military personnel stationed at altitude and humans on prolonged space expeditions in microgravity or on ground-based analog environments.

## Conclusions

Breathing stability is worsened after bed rest, throughout hypoxic exposure, and when bed rest and hypoxic stimuli are combined. These data are clinically relevant for patients who may be hypoxic and inactive (e.g., chronic obstructive pulmonary disorder). The symptoms of these patients represent a frequent cause of ICU admissions. Sleep architecture is affected in all trials, with a greater time spent in light sleep, consistent in each of the three test conditions after prolonged exposures. These results are clinically important for their implications in applying and enforcing strict sleep hygiene rules in ICU units, as well as planning of further treatment options for ICU-induced sleep disorders.

## Author contributions

OE, IM, and LD concept and research design; SM, DM, SK, and LD, performed experiments; SM, DM, SK, and LD analyzed data; SM and LD interpreted results; SM, DM, SK, and LD drafted manuscript; SM, DM, SK, OE, IM, and LD edited and revised manuscript; SM, DM, SK, OE, IM, and LD approved final version of manuscript.

### Conflict of interest statement

The authors declare that the research was conducted in the absence of any commercial or financial relationships that could be construed as a potential conflict of interest. The reviewer CAG and handling Editor declared their shared affiliation, and the handling Editor states that the process nevertheless met the standards of a fair and objective review.

## Abbreviations

AHI: apnea-hypopnea index
AMS: acute mountain sickness
BDC: baseline data collection
F_I_O_2_: fraction of inspired O_2_
HAMB: hypoxic ambulatory confinement condition
HAPE: high-altitude pulmonary edema
HBR: hypoxic bed rest condition
HR: heart rate
HVR: hypoxic ventilatory response
LLS: Lake Louise Score
N1: non-REM Stage 1 light sleep
N2: non-REM Stage 2 light sleep
N3: non-REM Stage 3 slow wave sleep
NBR: normoxic bed rest condition
NREM: on-REM sleep
PSG: polysomnography
REC: recovery day, out of condition
R: rapid eye movement sleep
SpO_2_: peripheral oxyhemoglobin saturation
TST: total sleep time
WASO: wake after sleep onset.
